# Building the infrastructure to make science metrics more scientific

**DOI:** 10.12688/f1000research.10422.2

**Published:** 2017-04-07

**Authors:** Jennifer Lin, Fiona L. Murphy, Mike Taylor, Liz Allen

**Affiliations:** 1 Department of Product Development, Crossref, Oxford, UK; 2 Associate Fellow, Institute for Environmental Analytics, University of Reading, Reading, UK; 3 Department of Research Metrics, Digital Science, London, UK; 4 Department of Strategic Initiatives, F1000, London, UK; 5 Visiting Senior Research Fellow, King's College London, London, UK

**Keywords:** Altmetrics, scientometrics, science policy, research indicators, research evaluation, impact, research policy, funding

## Abstract

Research leaders, policy makers and science strategists need evidence to support decision-making around research funding investment, policy and strategy.  In recent years there has been a rapid expansion in the data sources available that shed light onto aspects of research quality, excellence, use, re-use and attention, and engagement. This is at a time when the modes and routes to share and communicate research findings and data are also changing.

In this opinion piece, we outline a series of considerations and interventions that are needed to ensure that research metric development is accompanied by appropriate scrutiny and governance, to properly support the needs of research assessors and decision-makers, while securing the confidence of the research community. Key among these are: agreed ‘gold standards’ around datasets and methodologies; full transparency around the calculation and derivation of research-related indicators; and a strategy and roadmap to take the discipline of scientific indicators and research assessment to a more robust and sustainable place.

## Introduction

It is an exciting and challenging time for research evaluators and strategists; in the post-digital era, technical limitations around what can be used to assess different aspects of research are falling away. The availability of article-based citation metrics and indicators that capture research article reach, attention, and engagement is helping to reduce a reliance on misleading journal-based assumptions of scientific quality and importance. Many researchers now openly share components of their research – often within a research article, but increasingly outwith. For example, databases, datasets, software, and artistic outputs are often now on a range of platforms (e.g. Figshare, Zenodo) and independently citable (through the use of a digital identifier, such as a DOI). In addition, many researchers share analysis through non-traditional media (e.g. preprints, blog posts and policy documents).

At their essence, research metrics are designed to shed light on a range of attributes of research to support decision-making around resource allocation and research funding strategy (including tenure, career appointments and grant applications). In addition, metrics today routinely support national research assessment exercises, as exemplified by REF2014 in the UK and ERC2015 in Australia. Despite this, there continues to be limited investment in either research on the quality and validity of the indicators or the governance and stewardship of the data upon which indicators are derived. 

Policy experts and researchers have long petitioned to make research metrics more robust, evidence-based and *scientific* ( Lane, 2010) and therefore acceptable to the community they are meant to serve. Recent analyses have also reported on the current limitations of research metrics, calling for more research on, and improvements in, the infrastructure to support science indicators ( Hicks *et al*., 2015; Wilsdon *et al*., 2015). The EU also recently issued a consultation to put ‘alternative’ metrics on firmer footing as part of its drive to encourage open science approaches and robust ways to evaluate research ( Amsterdam Call for Action on Open Science, 2016). However, the ‘science’ of research metrics (scientometrics) paradoxically remains an orphan discipline given that more effective and accurate science metrics could make science more effective.

## Building an evidence base for metrics

We are now at a pivotal point of the research indicator story where a political and administrative appetite for research metrics to build and sustain efficient and effective research systems co-exists with a burgeoning in the sources of intelligence about research outputs. What is needed to harness this momentum is cross-sector agreement on the next best steps and actions to make research metrics more robust, transparent and empowered to work for the whole research community. An important part of this is to make research indicators used, valued and acceptable to the research community for the purpose in which they have been designed. To date much of the debate around the challenge in using research metrics robustly has centred on the ease with which research-related metrics – and bibliometrics in particular – are gamed and suffer from Goodhart’s Law where “ *when a measure becomes a target, it ceases to be a good measure*” ( Elton, 2004) so that they become poor indicators of either productivity or research quality.

Several initiatives are underway whose aim is, at least in part, to consider how to improve the evidence base upon which science is evaluated and make science more effective (see for example, the EU Open Science Policy Platform, and the UK Forum for Responsible Research Metrics [announced in September 2016]). The key ways that such initiatives will be able to make a real difference, is four-fold. First, ensure active participation from across the whole scientific research community in a broad way to include researchers, institutions and funding agencies, alongside scientific publishers, learned societies and technology platform providers. Second, deliver a roadmap for the key requirements needed to build and assure quality science metrics for the benefit of science. This should include consideration of how research productivity is best tracked and assessed, both quantitatively and qualitatively. Third, question existing assumptions around how we conduct and reward research, and test out new approaches and ways of working – and again considering how to incentivise the type of research that is required to deliver the required goals. Fourth, secure access to resources and influence, as well as make actionable decisions. 

Against this backdrop, we believe that there are now a number of very practical ingredients that can potentially act as part of a roadmap to ensure the development of robust and fair science indicators that have community support. We outline these below. 

### Definitions, descriptions and sources

For research metrics to be understood and used consistently there needs to be agreement around common vocabulary and descriptors of terms. As an example, CASRAI is building a dictionary of scholarly research output terminology. This dictionary has multiple users, including groups involved in the development of research metrics. 

The definitions themselves need to be definitive, openly sourced, managed, curated, versionable and quality assured. Additionally, the data upon which the indicator is best derived need to be identified. One of the challenges around research indicator derivation to date is that many of those in common usage are based upon opaque methodologies and proprietary datasets. This has eroded trust among the user base - many of whom don’t have access to the data - and pragmatically makes it difficult for particular metrics to be reproduced and explained. 

### Availability and preservation of Gold Standard (GS) data

An important concern around current research metrics is that they are often compiled and enabled through proprietary databases with locked access to the underlying data. This creates challenges for third parties wanting to replicate a metric, apply it in a different context or produce aggregate datasets from multiples sources. It also leads to mistrust and scepticism among users and those whose research is described ( Wilsdon *et al*., 2015).

The community needs a reference set – a Gold Standard (GS) dataset – for proper metrics development. A GS dataset would also enable an ongoing appraisal of best practice for a particular metric’s use and application – and potential inter-relationship with other metrics. Currently, a wide array of metrics is available. These make similar claims, but derive from different formulations. If enabled to work by correlating against a GS dataset, analysts can conduct systematic and rigorous testing and benchmarking for these options to surface the ones most useful across different applications. In short, while the open availability of raw metrics data is critical to transparency and to support innovation in metrics development and provisioning, we need a separate reference dataset that ensures the raw data which underlie a specific metric or metrics are properly preserved and audited.

### Towards open standards

In addition to the raw data, required analytical tools also need to be made available for true transparency and reproducibility (and thereby trust in the metrics). This includes products, such as a defined (minimum core) dataset, and open source standards on how the data are derived and defined (perhaps through an intermediary such as Crossref or by a cross-functional stakeholder group). The National Information Standards Organization’s work in this area can be built upon in future research. Commercial entities might also serve as potential sources where available to the broader community.

### Research on research metrics and scientific indicators

Perhaps most importantly given the stakes involved, we need greater consensus around how science and research-related metrics are best used to support decision making in science. As noted earlier, metrics need to be created to answer specific research evaluation questions – and where possible be able to avoid the potential to be gamed. Research on research (science of science) is needed to help answer the important research evaluation questions and determine which metrics are useful and have the potential to provide insight to these research questions. As researchers adopt new ways to share and publish their research at speed, metrics and indicators that track and assess the value, quality and utility of those activities need to keep pace.

We see a valuable role for funders to play in supporting this particular research area. The community working in the field is small and funding can be difficult to allocate even where funding for research evaluation studies is available (such as the UK’s Medical Research Council’s report on how science is funded). Focused funding is also needed to train a cadre of researchers to conduct experiments around what works for science and research, and this includes analyses of research assessment and metrics. Additionally, they (along with policy-makers) can contribute use cases and research questions to those developing metrics to ensure that the outputs are practical and meet real needs. Simply by taking additional notice of this field, funders will be making a critical contribution towards highlighting its significance and expediting progress. Having key leverage on the drivers, incentives and value systems of the research ecosystem, they can enable a shift in behaviours and culture.

Perhaps most importantly, it is paramount that and funding agencies and research institutions alike, work together to champion and incentivise the types of research and researcher behaviours that are likely to bring about desired outcomes and impacts – however wide the range of these might be. And, this might, interestingly, include de-emphasizing output in favour of seeking out more qualitative ways of assessing research ( Edwards & Roy, 2017).

### Investing in the online & digital infrastructure

As noted in Wilsdon *et al*., 2015, the digital infrastructure underpins not only the research enterprise but also the creation of metrics. Scholarly outputs of all stripes – articles, pre-prints, datasets, software, and peer review reports – need identifiers (such as DOIs) within this networked ecosystem to facilitate the derivation of metrics. This need extends beyond research artefacts: identifiers for researchers ( ORCIDs), funders ( Open Funder Registry), as well as research institutions. For research metrics to be open, trusted and useful, research objects need to be reliably and meaningfully linked to each other, as well as to researchers, institutions and funding agencies to support strategy and decision-making (see for example Amsterdam Call for Action on Open Science, 2016).

### Community memory on metrics development

Currently, research and documentation on metrics is dispersed. As a non-disciplinary grouping, not a single scholarly community or society spans all the relevant groups working on theory, advancing analytics, data quality, visualisation, policy (and economics). No single party takes responsibility for collecting or documenting process, evidence of good or bad practice, or any other significant issues. The value of these resources may not be immediately obvious, but their absence can stunt the progress of metrics utility, innovation, transparency and dependability.

## Moving forward: a path to fulfil these needs

As researchers adopt new ways to share their scholarly contributions at speed, metrics which describe and provide insight into that work need to keep pace. Different metrics are likely to have different value across output types, research fields and in different circumstances. Yet we believe that a coordinated, cross community effort to enhance our knowledge and application of research metrics is both timely and sensible.

At the time of writing, we welcomed the work being initiated by the EU Consultation on Metrics and the announcement of the UK’s Responsible Metrics Forum, both which aim to some degree to rethink the scope and use of research metrics. However, we would like to see the discourse move far beyond descriptors of the challenges of *using* current research metrics responsibly, to one that helps the research community to build research assessment into a discipline that can actively support efficiency in science and research. And one that starts to take practical steps to build the infrastructure to support research assessment and develop indicators.

To facilitate this, we recommend the establishment of a cross sector, community entity to be charged with building critical mass and momentum around research assessment and associated indicators/metrics. We envisage that there might be a number of guises that a cross community entity or effort could assume, including:

**1.**  an **independent non-profit membership organisation** (e.g. like ORCID) managed by a cross-sector board and executive.
**2.**  an **independent research metrics/indicators foundation** – funded by a consortium of national and independent research funding agencies, whose aim would be to deliver establishment of
**3.**  **an independent, international ‘office’ of research metrics/indicators** - funded by national governments and organisations, whose remit would be to develop standards and deliver research metrics – including to provide ‘ a Frascati Manual’ of definitions and standards for research/science metrics. This could include an ongoing programme of research (including ability to commission research) to keep pace with developments in science and research practice.
**4.**  an **international, distributed hub of experts** (similar to a learned society) that could, for example, commission and that can both deliver and advise on scientific indicators and commission work or work with an existing independent funding agency to support a research programme.

Such an entity could be governed and directed by a collective of independent research funding agencies or institutions, though would by necessity be a collective of relevant bodies. Or it could be configured entirely differently. What is important is that research assessment remains integral to the research enterprise; what is also clear is that as a discipline, it remains in its infancy and that to move forward, requires a cross-sector, cross research community involvement and engagement. We welcome initiatives that seek to seriously forge such collaboration, take research assessment to a more robust and sustainable footing, and as part of this, can help to spear-head the development, transparency and safe-guarding of ‘scientometrics’– be these quantitative or qualitative.
