# Supplementary material for: Building the infrastructure to make science metrics more scientific
Source: F1000Res. 2017 Apr 7;5:2897. Originally published 2016 Dec 21. [Version 2] doi: 10.12688/f1000research.10422.2 (PMC5405792; doi:10.12688/f1000research.10422.2)
Supplement: Peer review history

# Peer review: Referee response for version 2

**DOI:** 10.5256/f1000research.12261.r21669

**Published:** 2017-04-24

**Authors:** Maria Nedeva

**Affiliations:** 1 Manchester Institute of Innovation Research (MIoIR), Alliance Manchester Business School (MBS), The University of Manchester, Manchester, UK

I believe that the authors have responded to the concerns raised appropriately and this should be indexed.

I have read this submission. I believe that I have an appropriate level of expertise to confirm that it is of an acceptable scientific standard.

# Peer review: Referee response for version 2

**DOI:** 10.5256/f1000research.12261.r21668

**Published:** 2017-04-19

**Authors:** David J. Currie

**Affiliations:** 1 Department of Biology, University of Ottawa, Ottawa, ON, Canada

The opinions expressed in this paper are reasonable enough. My main reservation is that those opinions don’t go far beyond common sense, but that it is not at all obvious how to carry out these principles. As I said in my first review, preaching to scientists about what they should be doing is not very useful. Leading the way would be useful, but this paper does not do that.

 The modified manuscript does nothing to address my earlier concerns. So, no, I would not amend my earlier recommendation. However, it is possible that my objection may be out-of-bounds, given the editorial policies of the journal with respect to this article type. If that is the case, then I have no further reservations about the manuscript.

I have read this submission. I believe that I have an appropriate level of expertise to confirm that it is of an acceptable scientific standard, however I have significant reservations, as outlined above.

# Peer review: Referee response for version 1

**DOI:** 10.5256/f1000research.11230.r19504

**Published:** 2017-02-13

**Authors:** Ivan Oransky, Alison Abritis

**Affiliations:** 1 Retraction Watch, The Center for Scientific Integrity, New York, NY, USA; 2 Retraction Watch, The Center For Scientific Integrity, New York, NY, USA

Thank you for the opportunity to review this manuscript. We should note, however, that given the nature of the manuscript, which is really an editorial, we are of the opinion that it probably does not require the kind of rigorous peer review usually applied to original research.

 The manuscript presents a good summary of many of the issues facing research into metrics, and offers a plan for addressing them, at a very high level. We would suggest a few additions for improvement: 

-  A nod to the fact that metrics can always be gamed, and that while making them more scientific could cut down on this risk, it will likely always be possible. It might also bear mentioning that no matter what metrics we end up with, they are no substitute for reading a particular paper. Put another way, metrics may be useful for certain things (eg large-scale productivity) but not others (eg quality).
- A more specific set of next steps. What first steps might funders, scientists, and administrators take? A systematic review and meta-analysis? A gathering to frame the questions and identify funding priorities? Etc. 

We have read this submission. We believe that we have an appropriate level of expertise to confirm that it is of an acceptable scientific standard, however we have significant reservations, as outlined above.

# Peer review: Referee response for version 1

**DOI:** 10.5256/f1000research.11230.r19300

**Published:** 2017-01-13

**Authors:** Maria Nedeva

**Affiliations:** 1 Manchester Institute of Innovation Research (MIoIR), Alliance Manchester Business School (MBS), The University of Manchester, Manchester, UK

I believe that the title is appropriate and the abstract captures the essence of this opinion piece. I’m emphasising that this is an opinion piece because judgement regarding sources and data would be very different. There is much that I like about this piece and one of the main things is that it puts out there a very important at present discussion: how we use indicators in research evaluation and how we can do this better (or at least in a way that doesn’t disadvantage the development of science). The authors are well informed about the state of play and have given serious consideration to what can be done.

 I also can see how the very practical proposals in this piece could be implemented and yield some results.

 My reservations are about the failure to reach beyond the ‘technical’ – this is very needed though probably outside of what the authors have set out to achieve here. This is why, I believe that this piece should be published and, possibly, scholars in the UK and beyond encouraged to take part in this kind of discussion.

 Hope this helps.

I have read this submission. I believe that I have an appropriate level of expertise to confirm that it is of an acceptable scientific standard, however I have significant reservations, as outlined above.

# Peer review: Referee response for version 1

**DOI:** 10.5256/f1000research.11230.r18877

**Published:** 2017-01-03

**Authors:** David J. Currie

**Affiliations:** 1 Department of Biology, University of Ottawa, Ottawa, ON, Canada

In an opinion piece, Lin, Murphy, Taylor and Allen point out that: 1) researchers’ contributions to their fields are evaluated for purposes of research grant allocations, career advancement, prizes, etc.; and 2) the quality and validity of metrics that underlie these decisions are not well studied.

 Lin and colleagues call for the development of a discipline that will improve the evidence and infrastructure with which science is evaluated. Their point is well-taken. The manuscript was useful (to me) in pointing out some references and links to initiatives that are now underway in this field. However, to tell the reader what needs to be done is much less useful than actually doing something. This manuscript offers some reasonable suggestions about steps that might improve the evaluation of science; the difficulty is that the article does not present any evidence of an advance. More than opinion is necessary to advance the field.

I have read this submission. I believe that I have an appropriate level of expertise to confirm that it is of an acceptable scientific standard, however I have significant reservations, as outlined above.
